# The Effects of Skin-to-Skin Contact on Temperature and Breastfeeding Successfulness in Full-Term Newborns after Cesarean Delivery

**DOI:** 10.1155/2014/846486

**Published:** 2014-12-25

**Authors:** Shourangiz Beiranvand, Fatemeh Valizadeh, Reza Hosseinabadi, Yadollah Pournia

**Affiliations:** ^1^Faculty of Nursing, School of Nursing and Midwifery, Lorestan University of Medical Sciences, Khorramabad, Iran; ^2^Faculty of Nursing, Jondishapour Ahvaz University of Medical Sciences, Ahvaz, Iran; ^3^Faculty of Nursing, Social Determinants of Health Research Center, Lorestan University of Medical Sciences, Khorramabad, Iran; ^4^Faculty of Medicine, School of Medicine, Lorestan University of Medical Sciences, Khorramabad, Iran

## Abstract

*Background*. The skin-to-skin contact (SSC) of mother and newborn is uncommon full-term newborns after delivering via cesarean section due to the possibility of hypothermia in the infants. The aim of this study was to compare mothers' and infant's temperatures after delivering via cesarean section. *Material and Methods*. In this randomized clinical trial, 90 infant/mothers dyads delivered via cesarean section were randomized to SSC (*n* = 46) and routine care (*n* = 44). In experimental group, skin-to-skin contact was performed for one hour and in the routine group the infant was dressed and put in the cot according to hospital routine care. The newborns' mothers' temperatures in both groups were taken at half-hour intervals. The data was analyzed using descriptive statistics, *t*-tests, and chi-square tests. *Results*. The means of the newborns' temperatures immediately after SSC (*P* = 0.86), half an hour (*P* = 0.31), and one hour (*P* = 0.52) after the intervention did not show statistically significant differences between the two groups. The mean scores of the infants' breastfeeding assessment in SSC (8.76±3.63) and routine care (7.25±3.5) groups did not show significant differences (*P* = 0.048). *Conclusion*. Mother and infant's skin-to-skin contact is possible after delivering via cesarean section and does not increase the risk of hypothermia.

## 1. Introduction

One of the most important needs of infants at birth is the maintenance of temperature because an infant is not able to generate heat due to lack of shivering mechanism, and this leads to a rapid decline in its temperature [1]. Currently, the routine care to prevent hypothermia is to put the infant under a warmer, causing the separation of the mother and newborn. One of the most important roles of a nurse is to facilitate a close bonding relationship between the mother and infant. To fulfill this role and to treat hypothermia, nurses apply an efficient, accessible, and applicable method called mother and newborn skin-to-skin contact [2]. The movement of the infant's hands over the mother breasts in kangaroo care leads to increased secretion of oxytocin, which results in increased secretion of breast milk and breast heat. The heat is transferred from the mother to the baby because the mother's body temperature activates the baby's sensory nerves, and it results in the baby's relaxation, reduction in the tone of the sympathetic nerves, dilation of the skin vessels, and increase in the baby's body temperature [3].

Mother and infant skin-to-skin contact is well-known in full-term infants with natural deliveries [4]. However, it is believed that infants delivered via cesarean section are predisposed to hypothermia due to low temperature in the operation room, mother's unconsciousness, spread of mother's heat from the center to the environment, and reduction in mother's central temperature. Therefore, mother and infant skin-to-skin contact is potentially limited in infants after born via cesarean deliveries [5]. However, cesarean section surgery has increased dramatically, so that it has increased four times over the last 30 years [6]. The statistics by the Iranian Ministry of Health and Medical Education in 2004 obtained from different areas in Iran reported an estimation of 40 to 60% of caesarean sections in the country. Numerous studies have been conducted on the physiological effects of mother's skin-to-skin contact with preterm and full-term infants after natural deliveries, but few studies have been done on full-term newborns after being delivered via cesarean section [7].

Although mother and infant skin-to-skin contact immediately after birth and the start of breastfeeding during the first hour of birth are considered the top fourth measures to obtain baby friendly status within hospitals, these measures are still not done effectively in Iran and there has been no endeavor to initiate SSC in the country so that the mean of breastfeeding starting is 3.5 hours in natural deliveries and 6.9 hours in delivering via cesarean section, and only 1.5% of infants are breastfed during the first hour of birth [8]. According to the Center for Disease Prevention, only 32 percent of American's hospitals were doing skin-to-skin contact of mother and newborn for two hours after birth and the usual method is to take the baby under a radiant heating in the operating room and nursery word while the World Health Organization recommends that skin-to-skin contact for mothers and newborn should be done regardless of age, birth weight, and the clinical status [9]. The results of a study by Huang et al. (2006) showed that cesarean infants in skin-to-skin contact group had higher mean temperatures compared to the controls who received the routine care under the warmer [10]. Therefore, this study was conducted to determine the effect of skin-to-skin contact on infants' temperatures and breastfeeding successfulness in full-term infants after delivering via cesarean section.

## 2. Materials and Methods 

This study was designed as a Randomized Clinical Trial (RCT) conducted in Asali Hospital (west of Iran). The inclusion criteria for the mothers included singleton pregnancy, gestational age of 38–42 weeks, age range of 18–40 years old, and elective cesarean section surgery under spinal anesthesia. The exclusion criteria for the mothers included problems such as severe bleeding, uterine inertia, gestational diabetes and hypertension, and heart disease. The inclusion criteria for the infants included being full-term and having first- and fifth-minute Apgar above seven. The study excluded the infants with high risks and abnormalities or any other problems which needed hospitalization, according to the doctor's advice. According to sample size formula and considering the loss of samples 96 mother-infant dyads were selected. Then the samples were randomized to skin-to-skin (*n* = 48) and routine care (*n* = 48) groups via a random number table.

The data collection tools consisted of four parts. The first part included the demographic data of the mothers including age, weight, and gestational age, number of miscarriages, number of children, history of lactation, and history of problems during pregnancy, along with the demographic data of the infants including height, weight, head circumference, chest circumference, and first and fifth-minute Apgar. The second part was a form to record temperatures of the mothers and infants infrared ray thermometer on the forehead, IN4K8210 model. The third part was the standard Infant Breastfeeding Assessment Tool (IBAT), which included four subscales of sucking, rooting, readiness, and latching, each having 0–3 scores and 0–12 scores in total, which were completed through direct observation of infant breastfeeding. IBAT is a reliable tool for assessing infant success in first breastfeeding which has been used in several studies [11, 12]. The questionnaire was prepared from authentic scientific resources. Its validity was confirmed through the content validity based on the expert viewpoints of faculty members in the Faculty of Nursing and Midwifery in Khorramabad (west of Iran), and its reliability was confirmed through direct observation in a random sample of 20 cases of breastfeeding in the pilot study in which correlation coefficient was 0.95.

The fourth part was a form of maternal satisfaction with skin-to-skin contact, which consisted of 11 questions (yes, no, and not know) and was completed as a self-report with a confirmed validity in previous studies.

To perform the study, the researcher first arranged with the authorities of the Obstetrics Ward of Asalaian Hospital of Lorestan University of Medical Sciences (west of Iran) and then attended the hospital. The samples were selected from July 2011 to September 2011, according to the inclusion criteria of the study and their informed consent to participate in the study. Subsequently, the mothers' temperatures in the two groups before and after of surgery were recorded via the forehead infrared thermometer; infants temperatures were recorded on arrival at the operating room after their umbilical cords were cut and a general assessment of the infants then their first and fifth-minute Apgar scores were measured. The infants were then wrapped in blankets and taken to the nursery ward. In this unit, their temperatures were recorded after anthropometric measurements and vitamin K injections were performed. Then the babies were delivered to their mothers when mother back from operation room, and in the experimental group the naked infants in diapers were positioned between their mother breasts in a prone position. To preserve the infants' temperatures, their heads were covered with hats and their backs with suitable covers. The mothers and infants' temperatures were measured and recorded at the start of skin-to-skin contact, half an hour, and one hour after with infrared ray thermometer on the forehead. In the control group, the infants were dressed and embraced by their mothers to be breastfed, and their temperatures were measured at the time they were delivered to their mothers, after half an hour, and after one hour. Also, the mothers in the two groups were trained how to breastfeed. Then the Infant Breastfeeding Assessment Tool (IBAT) was applied to assess breastfeeding in the two groups, and the satisfaction form was completed by the mothers in the skin-to-skin contact group. Moreover, the temperatures of the nursery ward, the operation room, and the mother's ward were measured and recorded in all the steps.

To analyze the data, descriptive statistics including frequencies, means, standard deviations,* t*-tests, chi-square tests, and Kolmogorv-Smirnov tests were applied. The analyses were done and considered as blind and the analyst of the data was not aware of the classifications. To follow the ethical considerations, the researchers provided the participants with necessary information, and the secrecy of their personal data was followed. Therefore, the participants' names were not recorded in the forms. The study was approved by the Ethics Committee of Lorestan University of Medical Sciences (west of Iran).

## 3. Results

90 infant/mother dyads completed the study. Two infant/mother dyads in the skin-to-skin group and 4 infant/mother dyads in the routine group were excluded from the study due to neonatal respiratory distress syndrome.

The results showed no significant differences between the two groups concerning the means and standard deviations of the mothers' demographic data including age, weight, and gestational age, along with the infants' data including height, weight, head circumference, chest circumference, and first and fifth-minute Apgar. Moreover, the results of chi-square tests showed no significant differences between the two groups in terms of mother's educational level, number of miscarriages, number of children, history of lactation, history of problems during pregnancy, and infant's gender (Table 1).

The results also showed no significant differences between the two groups concerning the means and standard deviations related to mother's temperatures before and after the surgery, infant's temperatures on arrival at the operating room and the nursery room, and temperatures of the operation room and mother's and infants' rooms after arriving at the ward (Table 2).

In addition, the analysis of the data concerning the means and standard deviations of mother and infant's temperatures at the start of skin-to-skin contact, half an hour, and one hour after the contact did not show significant differences between the two groups of skin-to-skin and routine care (Table 3).

In general, repeated measure tests showed that the infants' temperatures repeated over time, namely, at the start of skin-to-skin contact, half an hour, and one hour after the contact, did not show significant differences in the two groups of skin-to-skin contact (*P* = 0.201, *F* = 1.68) and routine care (*P* = 0.4, *F* = 0.59).

Moreover, repeated measure tests showed significant differences in terms of the mothers' temperatures in the skin-to-skin contact group (*P* = 0.01, *F* = 0.002), but the differences in the routine care group were not statistically significant (*P* = 0.11, *F* = 2.74). The disturbing effects of maternal and neonatal temperatures before the intervention were not significant in any of the cases (*P* > 0.05).

Regarding the breastfeeding assessment of the infants after delivered via cesarean section, 52.2% of the infants in the skin-to-skin contact group and 25% in the routine care group showed readiness to breastfeed without doing any attempts, and the chi-square test showed the difference between the two groups to be statistically significant (*P* = 0.021).

In terms of sucking, 50% and 37% of the infants in the skin-to-skin contact group showed good and moderate sucking, respectively. In the routine care group, 36.4% and 27.3% of the infants showed good and moderate sucking, respectively. The chi-square test showed statistically significant differences (*P* = 0.03).

Concerning latching, 39/1% of the infants in the skin-to-skin contact group and 20/5% in the routine care group held mothers' breasts immediately, and chi-square test did not show a statistically significant difference between the groups (*P* = 0.21).

In relation to rooting, 47.8% in the skin-to-skin contact group and 29.5% the routine care group, respectively, immediately started looking for mothers' breasts to suck, and the chi-square test did not show significant difference between the two groups (*P* = 0.19) (Table 4).

The means and standard deviations of the total score of breastfeeding assessment related to the infants in the skin-to-skin contact group and in the routine care group were 8.76 ± 3.63 and 7.25 ± 3.5, respectively, and the* t*-test did not show a significant difference between the two groups (*P* = 0.048). Most of the mothers gave “yes” answers to the questions related to their satisfaction with skin-to-skin contact.

## 4. Discussion

The findings of the present study showed that skin-to-skin contact between mother and infant after delivering via cesarean section did not cause a drop in infants' temperatures and that it was effective in their breastfeeding successfulness. Gouchon et al., 2010, compared infants' temperatures after delivering via cesarean section in two groups of skin-to-skin and routine care. They measured infants' temperatures every half an hour for two hours after skin-to-skin contact, and did not report significant difference between the two groups. In their study, the infants in the skin-to-skin group started the initial breastfeeding sooner than the infants in the routine care group [5].

Infant skin temperature rises after birth in relation to an increased metabolic rate and the mechanism by which skin-to-skin contact influences the infant's temperature is not fully known. It has been suggested that the touch, light pressure, and, in particular, the warmth received by the infant during the skin-to-skin contact activate sensory nerves, leading to cutaneous vasodilation and increased skin temperature. The increase in maternal breast skin temperature occurring in the postpartum period is probably on expression of an inborn psychophysiological pattern which aimed at helping the mother in her interaction with the baby after birth. The maternal giving of warmth diminishes the risk for development of hypothermia in the infant in the postpartum period [13].

Chiu and Anderson (2009) also investigated newborns' temperatures with breastfeeding difficulties during skin-to-skin contact with their mothers and found that the newborns' temperatures reached the normal rate of 36.5 to 37.6° Celsius and became fixed at that level, showing that mothers were able to regulate the newborns' temperatures in kangaroo care [14].

A study by Keshavarz and Haghighi which investigated the effects of kangaroo contact on physiological variables in term neonates after cesarean section included 160 neonates and assigned them randomly to skin-to-skin contact and routine care groups. The newborns' temperatures in the skin-to-skin group were measured half an hour and an hour after the contact and half an hour after the cessation of contact. Moreover, the newborns' temperatures in the routine care group were measured half an hour, an hour, and one and a half hour after they were placed in cots. The mean temperature in the skin-to-skin group was 36.8 compared to the mean temperature of 36.6 in the routine care group (*P* = 0.05), and the mean temperature one hour after skin-to-skin contact was 36.9, which was higher than the mean temperature of 36.6 in the control group (*P* = 0.001). In total, the mothers in the skin-to-skin contact group had more satisfaction [15]. These results are consistent with the results of our study. In these studies that were conducted, mothers' temperatures were not measured but in our study were measured and did not show significant differences between the two groups of skin-to-skin and routine care.

This study showed that infants in the skin-to-skin group were more successful in rooting reflex and the scores of the investigated breastfeeding indicators were higher in the skin-to-skin contact group. In the study of Bystrova et al., 2009, the infants in skin-to-skin contact with their mothers after birth soon started to search for the mother's breast, found the breast, and started sucking without the help of the mother or care staff [16].

Also infants sucking reflexes the scores of the investigated breastfeeding indicators that were higher in the skin-to-skin contact group. Some studies have shown that term newborns placed skin to-skin on the mother's abdomen immediately after birth spontaneously start moving toward the breasts and onto the nipple, but these behaviors are less likely to occur if infants are first placed under a radiant warmer [17].

Concerning latching, although there was not a statistically significant difference between the two groups, greater number of the infants in the skin-to-skin contact group held mothers' breasts immediately. In the first hours postpartum, the mother and infant learn to breastfeed together. In the first phase called self-attached breastfeeding, the baby latches to the breast without assistance and self-attaches to the breast using the stepping-crawling reflex. In the second phase, called collaborative breastfeeding, the mother and baby work together to achieve the latch and feeding. If the infant has been placed skin-to-skin without separation until self-attachment has been accomplished, although each type of latch and suckling deviation from optimal is associated with maternal pain, beginning to breastfeed without prefeeding behavior in the infant is associated with the highest levels of pain [18].

In this study breastfeeding successfulness in the skin-to-skin contact group was higher than in the routine care group. Moore and Anderson in their study reported stronger readiness, sucking, and quicker readiness in the infants in the skin-to-skin contact group compared to those in the routine care group [11]. Khadivzadeh and Karimi (2009) also reported higher breastfeeding successfulness in the skin-to-skin contact group compared to the routine care group [19].

Newborn babies should not be separated from their mothers except for significant medical reasons but should be placed skin-to-skin as soon as possible after birth to have the ability to start self-regulation [16].

In the present study, most of the mothers were satisfied with the skin-to-skin contact with their infants and were willing to continue this type of care. In a study by Carfoot et al. (2005) 90% of the mothers were satisfied with mother and neonate skin-to-skin contact and 86% were willing to continue the care in the future. Moreover, 90% and 81% of the infants, in the skin-to-skin contact and the routine care groups, respectively, had successful breastfeeding, showing no significant differences between the groups [12].

This care was performed for the first time in Asali Hospital of Khorramabad (west of Iran). This method of caring is simple, safe, economical, practical, and applicable. It is also a therapeutic method to prevent hypothermia in term neonates. Therefore, it is necessary to standardize nursing workforce to implement this method routinely after cesarean sections. To implement the method, it is recommended that extensive research on it should be performed.

## 5. Conclusion

According to the results of the present study, it is concluded that skin-to-skin contact after delivering via cesarean section was possible, maternal satisfaction level with skin-to-skin contact was higher, and cesarean neonates were not prone to hypothermia and improve breastfeeding initiation and facilitate the first successful experience of breastfeeding compared to routine method of infant care in delivered via cesarean section. The results of this study can be applied in various areas of nursing including nursing management, nursing education, and nursing research.

## Figures and Tables

**Table 1:** The relationship between demographic characteristics of mothers and infants in the skin-to-skin contact and routine care groups.

Variable	Group		*P*	*t*
	Skin-to-skin contact	Routine care		
Mother's age	27.66 ± 9.32	27.28 ± 6.88	0.83	0.22
Mother's weight (kg)	79 ± 12.95	81.3 ± 11.73	0.37	−0.9
Mother's gestational age (week)	39 ± 0.92	38.8 ± 1.017	0.66	0.44
Infant's weight	3240 ± 287.8	3220 ± 339.9	0.78	0.28
Infant's head circumference (cm)	35.5 ± 1.03	35.7 ± 1.12	0.42	−0.8
Infant's chest circumference (cm)	34.2 ± 1.07	34.3 ± 1.3	0.6	−5.3
Infant's height (cm)	49.8 ± 3.52	49.8 ± 3.10	0.83	−0.2
First-minute Apgar	9 ± 0	9 ± 0	—	—
Fifth-minute Apgar	10 ± 0	10 ± 0	—	—
Number of miscarriages				
0	39 (84.8%)	39 (88.6%)	0.83	*χ* ^2^ = 0.35
1	6 (13%)	4 (9.1%)		
2	1 (2.2%)	1 (2.3%)		
Number of pregnancies				
1, 2	37 (73%)	37 (79.5%)	0.67	*χ* ^2^ = 3.2
3 and more	9 (26.1%)	8 (20.5%)		
Number of children				
1	23 (50%)	27 (61.4%)	0.27	*χ* ^2^ = 1.2
More than 1	23 (50%)	17 (38.8%)		
Educational level				
Lower than high school diploma	19 (41.3%)	13 (29.5)	0.59	*χ* ^2^ = 1.92
High school diploma	22 (48.8%)	23 (52.3%)		
Bachelor's degree and higher	5 (10.9%)	8 (18.2%)		
Infant's gender				
Female	23 (50%)	26 (59.1%)	0.38	*χ* ^2^ = 0.75
Male	23 (50%)	18 (40.9%)		
History of lactation				
Yes	22 (48.8%)	13 (29.5%)	0.14	*χ* ^2^ = 3.8
No	24 (52.2%)	31 (70.5%)		
Problems during pregnancy				
Yes	26 (56.5%)	27 (61.4%)	0.64	*χ* ^2^ = 0.22
No	20 (43.5%)	17 (38.6%)		

**Table 2:** Comparison of the means and standard deviations related to the general situations of the mothers and infants in the skin-to-skin contact and routine care groups.

Variable	Group		*t*	*P*
	Skin-to-skin contact	Routine care		
Preoperative maternal temperature	36.44 ± 0.38	36.42 ± 0.48	0.13	0.9
Postoperative maternal temperature	36.48 ± 0.37	36.4 ± 0.41	0.98	0.32
Infant's temperature in the operating room	36.2 ± 0.62	36 ± 0.58	0.83	0.4
Infant's temperature on arrival at the nursery unit	36.7 ± 0.4	36.8 ± 0.5	−1.6	0.11
Temperature of the operating room	28 ± 1.2	28 ± 1.52	0.3	0.75
Temperature of the nursery unit	28.1 ± 0.96	28 ± 0.61	0.035	0.97
Temperature of the mother's room	29.9 ± 0.64	29.7 ± 1.63	0.8	0.42

**Table 3:** Comparison of the means and standard deviations related to the mothers and infants' temperatures in the skin-to-skin contact and routine care group.

Variable	Group		*t*	*P*
	Skin-to-skin contact	Routine care		
Maternal temperature at the start of the intervention	36.56 ± 0.46	36.4 ± 0.41	1.6	0.11
Maternal temperature half an hour after the intervention	36.56 ± 0.46	36.4 ± 0.52	1.6	0.11
Maternal temperature one hour after the intervention	36.43 ± 0.46	36.2 ± 0.67	1.6	0.11
Infant's temperature at the start of the intervention	36.35 ± 0.46	36.32 ± 0.46	0.16	0.86
Infant's temperature half an hour after the intervention	36.25 ± 0.5	36.4 ± 0.48	−1.01	0.31
Infant's temperature one hour after the intervention	36.44 ± 0.45	36.4 ± 0.48	0.63	0.52

**Table 4:** Breastfeeding assessment of the cesarean infants in the skin-to-skin contact and routine care groups.

Variable	Group		*χ* ^2^	*P*
	Skin-to-skin contact	Routine care		
Readiness				
With no attempts	24 (52.2%)	11 (25%)	9.68	0.021
Needing weak stimulation	13 (28.3%)	19 (43.2%)		
Needing more stimulation	4 (8.7%)	11 (25%)		
Sleepiness	5 (10.9%)	3 (6.8%)		
Sucking				
Good	23 (50%)	16 (36.4%)	8.42	0.03
Moderate	17 (37%)	12 (27.3%)		
Weak	2 (4.3%)	11 (25%)		
No sucking	4 (8.7%)	5 (11.4%)		
Latching				
Immediately	18 (39.1%)	9 (20.5%)	4.44	0.21
After 3–10 minutes	15 (32.6%)	15 (34.1%)		
After more than 10 minutes	9 (19.6%)	14 (31.8%)		
Not start breastfeeding	4 (8.7%)	6 (13.6%)		
Rooting				
Immediately	22 (47.8%)	13 (29.5%)	4.68	0.19
Needing stimulation	17 (37%)	18 (40.9%)		
Weak rooting	3 (6.5%)	8 (18.2%)		
No rooting	4 (8.7%)	5 (11.4%)		
